# *catena*-Poly[[[aqua­tris­(pyridine-κ*N*)nickel(II)]-μ-2,3,5,6-tetra­chloro­benzene-1,4-dicarboxyl­ato-κ^2^ *O* ^1^:*O* ^4^] pyridine monosolvate]

**DOI:** 10.1107/S1600536810045794

**Published:** 2010-11-20

**Authors:** Chang-Ge Zheng, Pei-Pei Zhang, Peng Zhang, Song Li

**Affiliations:** a School of Chemical and Material Engineering, Jiangnan University, 1800 Lihu Road, Wuxi, Jiangsu Province 214122, People’s Republic of China

## Abstract

The asymmetric unit of the title compound, {[Ni(C_8_Cl_4_O_4_)(C_5_H_5_N)_3_(H_2_O)]·C_5_H_5_N}_*n*_, contains two independent nickel(II) cations displaying a distorted octa­hedral coordination geometry provided by the N atoms of three pyridine mol­ecules, the O atom of a water mol­ecule, and O atoms of two monodentate μ_2_-bridging tetra­chloro­terephthalate dianions. The metal atoms are linked by the dianions into zigzag chains running parallel to [11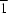]. The crystal packing is stabilized by O—H⋯N and O—H⋯O hydrogen bonds.

## Related literature

For the modelling of hydrogen adsorption in metal-organic frameworks, see: Mulder *et al.* (2005 ▶); Zheng *et al.* (2009 ▶). For related structures, see: Kim *et al.* (2003 ▶); Go *et al.* (2004 ▶); Wang *et al.* (2003 ▶); Li *et al.* (2003 ▶); Zheng *et al.* (2008 ▶). 
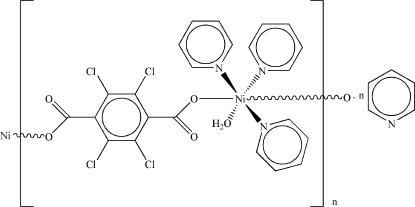


## Experimental

### 

#### Crystal data

[Ni(C_8_Cl_4_O_4_)(C_5_H_5_N)_3_(H_2_O)]·C_5_H_5_N
*M* *_r_* = 1390.01
Triclinic, 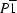

*a* = 8.6148 (6) Å
*b* = 17.6879 (10) Å
*c* = 21.0617 (10) Å
α = 68.279 (5)°
β = 79.750 (6)°
γ = 84.853 (6)°
*V* = 2932.9 (3) Å^3^
*Z* = 2
Mo *K*α radiation
μ = 1.07 mm^−1^
*T* = 293 K
0.26 × 0.21 × 0.10 mm

#### Data collection

Bruker APEXII diffractometer
Absorption correction: multi-scan (*SADABS*; Sheldrick, 2008*a* ▶) *T*_min_ = 0.688, *T*_max_ = 1.000
26508 measured reflections
11670 independent reflections
8092 reflections with *I* > 2σ(*I*)
*R*_int_ = 0.060

#### Refinement

*R*[*F* ^2^ > 2σ(*F* ^2^)] = 0.076
*wR*(*F* ^2^) = 0.151
*S* = 1.07
11670 reflections
757 parameters
H-atom parameters constrained
Δρ_max_ = 0.38 e Å^−3^
Δρ_min_ = −0.36 e Å^−3^

### 

Data collection: *APEX2* (Bruker, 2005 ▶); cell refinement: *SAINT* (Bruker, 2005 ▶); data reduction: *SAINT*; program(s) used to solve structure: *SHELXS97* (Sheldrick, 2008*b* ▶); program(s) used to refine structure: *SHELXL97* (Sheldrick, 2008*b* ▶); molecular graphics: *PLATON* (Spek, 2009 ▶); software used to prepare material for publication: *SHELXL97*.

## Supplementary Material

Crystal structure: contains datablocks global, I. DOI: 10.1107/S1600536810045794/rz2513sup1.cif

Structure factors: contains datablocks I. DOI: 10.1107/S1600536810045794/rz2513Isup2.hkl

Additional supplementary materials:  crystallographic information; 3D view; checkCIF report

## Figures and Tables

**Table 1:** Hydrogen-bond geometry (Å, °)

*D*—H⋯*A*	*D*—H	H⋯*A*	*D*⋯*A*	*D*—H⋯*A*
O9—H9*A*⋯O2	0.85	2.02	2.751 (4)	143
O9—H9*B*⋯N7	0.85	1.89	2.699 (6)	159
O10—H10*A*⋯N8^i^	0.85	1.97	2.783 (6)	161
O10—H10*B*⋯O3^ii^	0.85	1.83	2.677 (4)	174

Symmetry codes: (i) 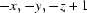; (ii) 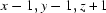.

**Fig. 1.**
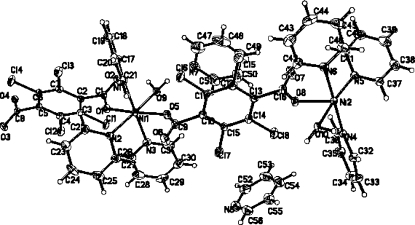
The asymmetric unit of the title complex showing 30% probability displacement ellipsoids and the atom-numbering scheme.

## supplementary crystallographic information

### Comment

Transition metal complexes have attracted considerable interest, owning to their elegant framework topologies as well as their potential applications in gas sorption, catalysis and optoelectronic devices, and a considerable amount of research work has been done on this type of complexes. However, there are few reports on polyhalogenated benzene-1,4-dicarboxylic ligands, especially tetrachloroterephthalic acid. Computer calculations have suggested that halogens such as chlorine can enhance the adsorption of hydrogen molecules in metal organic frameworks (Mulder *et al.*, 2005; Zheng *et al.*, 2009), so the title compound was synthesized and its crystal structure is reported herein.

The asymmetric unit of the title compound (Fig. 1) consists two independent nickel(II) cations having distorted octahedral coordination geometry, where the equatorial plane is provided by the N atoms of three pyridine molecules and the O atom of a water molecule, and the axial positions are occupied by the O atoms of two monodentate µ_2_-bridging tetrachloroterephthalate dianions. The bridging role of the dianions results in the formation of one-dimensional neutral zigzag chains running parallel to the [111] direction. The Ni—O bond lengths lie in the range 2.065 (3)–2.095 (3) Å, and agree well with the values reported in the literature for related compounds (Kim *et al.*, 2003; Go *et al.*, 2004). The Ni—N bond lengths lie in the range of 2.094 (4)–2.124 (4) Å, and are also comparable with those reported for the similar complexes (Wang *et al.*, 2003; Li *et al.*, 2003; Zheng *et al.*, 2008). The crystal packing is stabilized by O—H···N and O—H···O hydrogen interactions (Table 1).

### Experimental

All the reagents and solvents empolyed were commercially available. Tetrachloroterephthalic acid was purified by recrystallization. The title compound was synthesized by slow vapour diffusion at room temperature of pyridine (3 ml) in to a methanol solution (3 ml) containing a mixture of tetrachloroterephthalic acid (0.0304 g, 0.10 mmol) and NiCl_2_.6H_2_O (0.0476 g, 0.20 mmol) diluted with deionized water (2 ml). After ten days, green block-shaped crystals were obtained.The green block-shaped crystals were collected by filtration, washed with methanol (3 ml), and air dried to give the title complex (0.09 g, 65% yield). Elemental analysis (%) calcd. for C_56_H_44_Cl_8_N_8_Ni_2_: C, 48.34%; H, 3.17%; N, 8.06%; Found: C,48.14%; H, 2.98%; N, 7.94%.

### Refinement

All H atoms were placed in geometrically idealized positions and constrained to ride on their parent atoms with C—H = 0.93 Å, O—H = 0.85 Å, and with *U*_iso_(H) = 1.2 *U*_iso_(C) or 1.5 *U*_iso_(O).

### Crystal data

**Table:**

[Ni(C_8_Cl_4_O_4_)(C_5_H_5_N)_3_(H_2_O)]·C_5_H_5_N	*Z* = 2
*M**_r_* = 1390.01	*F*(000) = 1416
Triclinic, *P*1	*D*_x_ = 1.574 Mg m^−^^3^
Hall symbol: -P 1	Mo *K*α radiation, λ = 0.71073 Å
*a* = 8.6148 (6) Å	Cell parameters from 11475 reflections
*b* = 17.6879 (10) Å	θ = 3.0–27.5°
*c* = 21.0617 (10) Å	µ = 1.07 mm^−^^1^
α = 68.279 (5)°	*T* = 293 K
β = 79.750 (6)°	Block, green
γ = 84.853 (6)°	0.26 × 0.21 × 0.10 mm
*V* = 2932.9 (3) Å^3^	

### Data collection

**Table:**

Bruker APEXII diffractometer	11670 independent reflections
Radiation source: fine-focus sealed tube	8092 reflections with *I* > 2σ(*I*)
graphite	*R*_int_ = 0.060
Detector resolution: 28.5714 pixels mm^-1^	θ_max_ = 26.2°, θ_min_ = 3.0°
dtprofit.ref scans	*h* = −10→10
Absorption correction: multi-scan (*SADABS*; Sheldrick, 2008*a*)	*k* = −21→18
*T*_min_ = 0.688, *T*_max_ = 1.000	*l* = −26→25
26508 measured reflections	

### Refinement

**Table:**

Refinement on *F*^2^	Primary atom site location: structure-invariant direct methods
Least-squares matrix: full	Secondary atom site location: difference Fourier map
*R*[*F*^2^ > 2σ(*F*^2^)] = 0.076	Hydrogen site location: inferred from neighbouring sites
*wR*(*F*^2^) = 0.151	H-atom parameters constrained
*S* = 1.07	*w* = 1/[σ^2^(*F*_o_^2^) + (0.048*P*)^2^ + 1.8*P*] where *P* = (*F*_o_^2^ + 2*F*_c_^2^)/3
11670 reflections	(Δ/σ)_max_ < 0.001
757 parameters	Δρ_max_ = 0.38 e Å^−^^3^
0 restraints	Δρ_min_ = −0.36 e Å^−^^3^

### Special details

**Table:**

Geometry. All e.s.d.'s (except the e.s.d. in the dihedral angle between two l.s. planes) are estimated using the full covariance matrix. The cell e.s.d.'s are taken into account individually in the estimation of e.s.d.'s in distances, angles and torsion angles; correlations between e.s.d.'s in cell parameters are only used when they are defined by crystal symmetry. An approximate (isotropic) treatment of cell e.s.d.'s is used for estimating e.s.d.'s involving l.s. planes.
Refinement. Refinement of *F*^2^ against ALL reflections. The weighted *R*-factor *wR* and goodness of fit *S* are based on *F*^2^, conventional *R*-factors *R* are based on *F*, with *F* set to zero for negative *F*^2^. The threshold expression of *F*^2^ > σ(*F*^2^) is used only for calculating *R*-factors(gt) *etc*. and is not relevant to the choice of reflections for refinement. *R*-factors based on *F*^2^ are statistically about twice as large as those based on *F*, and *R*- factors based on ALL data will be even larger.

### Fractional atomic coordinates and isotropic or equivalent isotropic displacement parameters (Å^2^)

**Table:**

	*x*	*y*	*z*	*U*_iso_*/*U*_eq_	
Ni1	0.46067 (7)	0.47947 (3)	0.23768 (3)	0.02446 (16)	
Ni2	0.00680 (7)	0.00952 (3)	0.76709 (3)	0.02428 (16)	
Cl1	0.96838 (15)	0.62945 (8)	0.09662 (6)	0.0431 (3)	
Cl2	1.09020 (14)	0.74578 (8)	−0.05442 (6)	0.0412 (3)	
Cl3	0.37903 (14)	0.75032 (8)	0.06981 (6)	0.0417 (3)	
Cl4	0.50078 (15)	0.86514 (8)	−0.08212 (7)	0.0456 (4)	
Cl5	−0.09677 (17)	0.28669 (9)	0.57970 (7)	0.0513 (4)	
Cl6	−0.01812 (17)	0.37233 (9)	0.41947 (7)	0.0502 (4)	
Cl7	0.53227 (16)	0.20335 (9)	0.42029 (7)	0.0493 (4)	
Cl8	0.46070 (16)	0.12525 (9)	0.58077 (7)	0.0472 (4)	
O1	0.5606 (4)	0.57825 (18)	0.15395 (15)	0.0304 (8)	
O2	0.6420 (4)	0.66386 (19)	0.19776 (15)	0.0333 (8)	
O3	0.8211 (4)	0.8264 (2)	−0.18049 (15)	0.0354 (8)	
O4	0.9117 (4)	0.91544 (18)	−0.14307 (15)	0.0303 (8)	
O5	0.3744 (4)	0.38525 (19)	0.32687 (15)	0.0325 (8)	
O6	0.2378 (5)	0.2903 (2)	0.31443 (17)	0.0510 (11)	
O7	0.1898 (6)	0.2018 (3)	0.68576 (19)	0.0683 (14)	
O8	0.0846 (4)	0.09826 (19)	0.67262 (15)	0.0334 (8)	
O9	0.5614 (4)	0.51792 (19)	0.30244 (15)	0.0322 (8)	
H9A	0.5796	0.5685	0.2865	0.048*	
H9B	0.5209	0.5157	0.3429	0.048*	
O10	−0.0918 (4)	−0.04115 (19)	0.70833 (14)	0.0299 (8)	
H10A	−0.0499	−0.0479	0.6710	0.045*	
H10B	−0.1203	−0.0848	0.7418	0.045*	
N1	0.2520 (4)	0.5464 (2)	0.25037 (18)	0.0282 (9)	
N2	0.3805 (4)	0.4496 (2)	0.16131 (19)	0.0294 (9)	
N3	0.6745 (5)	0.4151 (2)	0.2259 (2)	0.0321 (10)	
N4	0.2190 (4)	−0.0563 (2)	0.75740 (18)	0.0252 (9)	
N5	0.0898 (4)	0.0484 (2)	0.83883 (18)	0.0280 (9)	
N6	−0.2077 (4)	0.0743 (2)	0.7758 (2)	0.0320 (10)	
N7	0.4136 (7)	0.4726 (3)	0.4346 (2)	0.0546 (14)	
N8	−0.1032 (6)	0.0804 (3)	0.3961 (2)	0.0498 (12)	
C1	0.6234 (5)	0.6413 (3)	0.1504 (2)	0.0263 (11)	
C2	0.6812 (5)	0.6979 (3)	0.0762 (2)	0.0251 (10)	
C3	0.8388 (5)	0.6961 (3)	0.0472 (2)	0.0265 (11)	
C4	0.8924 (5)	0.7469 (3)	−0.0203 (2)	0.0260 (10)	
C5	0.7873 (5)	0.7984 (3)	−0.0605 (2)	0.0251 (10)	
C6	0.6309 (5)	0.7999 (3)	−0.0318 (2)	0.0268 (11)	
C7	0.5768 (5)	0.7495 (3)	0.0357 (2)	0.0254 (10)	
C8	0.8435 (5)	0.8513 (3)	−0.1355 (2)	0.0258 (10)	
C9	0.2918 (6)	0.3232 (3)	0.3475 (2)	0.0324 (12)	
C10	0.2531 (5)	0.2835 (3)	0.4270 (2)	0.0276 (11)	
C11	0.1141 (5)	0.3009 (3)	0.4637 (2)	0.0289 (11)	
C12	0.0784 (6)	0.2628 (3)	0.5353 (2)	0.0306 (11)	
C13	0.1823 (6)	0.2071 (3)	0.5724 (2)	0.0289 (11)	
C14	0.3229 (6)	0.1906 (3)	0.5358 (2)	0.0326 (12)	
C15	0.3575 (5)	0.2272 (3)	0.4644 (2)	0.0293 (11)	
C16	0.1491 (6)	0.1666 (3)	0.6518 (2)	0.0347 (12)	
C17	0.2511 (6)	0.6220 (3)	0.2509 (2)	0.0341 (12)	
H17A	0.3472	0.6460	0.2444	0.041*	
C18	0.1151 (6)	0.6655 (3)	0.2606 (3)	0.0418 (13)	
H18A	0.1195	0.7173	0.2617	0.050*	
C19	−0.0277 (6)	0.6317 (4)	0.2687 (3)	0.0437 (14)	
H19A	−0.1214	0.6609	0.2739	0.052*	
C20	−0.0297 (6)	0.5538 (4)	0.2689 (2)	0.0437 (14)	
H20A	−0.1247	0.5289	0.2752	0.052*	
C21	0.1122 (6)	0.5135 (3)	0.2595 (2)	0.0337 (12)	
H21A	0.1105	0.4610	0.2596	0.040*	
C22	0.3325 (6)	0.5079 (3)	0.1062 (2)	0.0360 (12)	
H22A	0.3188	0.5606	0.1063	0.043*	
C23	0.3022 (7)	0.4942 (4)	0.0492 (3)	0.0518 (16)	
H23A	0.2699	0.5368	0.0119	0.062*	
C24	0.3207 (7)	0.4170 (4)	0.0488 (3)	0.0579 (18)	
H24A	0.3032	0.4058	0.0109	0.070*	
C25	0.3658 (7)	0.3562 (4)	0.1059 (3)	0.0552 (17)	
H25A	0.3783	0.3030	0.1071	0.066*	
C26	0.3921 (6)	0.3738 (3)	0.1606 (3)	0.0382 (13)	
H26A	0.4194	0.3314	0.1993	0.046*	
C27	0.7585 (7)	0.4243 (3)	0.1639 (3)	0.0450 (14)	
H27A	0.7183	0.4584	0.1246	0.054*	
C28	0.9025 (7)	0.3853 (4)	0.1559 (3)	0.0559 (16)	
H28A	0.9589	0.3936	0.1120	0.067*	
C29	0.9613 (7)	0.3341 (3)	0.2137 (4)	0.0533 (16)	
H29A	1.0573	0.3063	0.2096	0.064*	
C30	0.8770 (7)	0.3243 (3)	0.2773 (3)	0.0500 (15)	
H30A	0.9152	0.2906	0.3172	0.060*	
C31	0.7346 (6)	0.3652 (3)	0.2813 (3)	0.0391 (13)	
H31A	0.6771	0.3577	0.3249	0.047*	
C32	0.2256 (6)	−0.1339 (3)	0.7609 (2)	0.0317 (11)	
H32A	0.1318	−0.1612	0.7713	0.038*	
C33	0.3659 (6)	−0.1747 (3)	0.7497 (3)	0.0430 (14)	
H33A	0.3650	−0.2283	0.7523	0.052*	
C34	0.5064 (6)	−0.1364 (3)	0.7350 (3)	0.0419 (13)	
H34A	0.6020	−0.1629	0.7271	0.050*	
C35	0.5007 (6)	−0.0571 (3)	0.7322 (2)	0.0370 (13)	
H35A	0.5935	−0.0293	0.7233	0.044*	
C36	0.3577 (6)	−0.0196 (3)	0.7427 (2)	0.0302 (11)	
H36A	0.3565	0.0343	0.7395	0.036*	
C37	0.1480 (5)	−0.0066 (3)	0.8924 (2)	0.0326 (11)	
H37A	0.1647	−0.0597	0.8935	0.039*	
C38	0.1849 (6)	0.0113 (4)	0.9465 (3)	0.0470 (15)	
H38A	0.2262	−0.0288	0.9828	0.056*	
C39	0.1598 (7)	0.0884 (4)	0.9456 (3)	0.0600 (18)	
H39A	0.1815	0.1020	0.9817	0.072*	
C40	0.1015 (8)	0.1465 (4)	0.8899 (3)	0.0565 (17)	
H40A	0.0834	0.2000	0.8877	0.068*	
C41	0.0708 (6)	0.1239 (3)	0.8381 (3)	0.0394 (13)	
H41A	0.0345	0.1637	0.8002	0.047*	
C42	−0.2697 (6)	0.1214 (3)	0.7192 (3)	0.0379 (13)	
H42A	−0.2160	0.1244	0.6760	0.046*	
C43	−0.4082 (7)	0.1651 (4)	0.7225 (3)	0.0515 (16)	
H43A	−0.4466	0.1974	0.6821	0.062*	
C44	−0.4900 (8)	0.1609 (4)	0.7858 (4)	0.0615 (18)	
H44A	−0.5839	0.1906	0.7890	0.074*	
C45	−0.4310 (7)	0.1123 (4)	0.8442 (4)	0.0597 (17)	
H45A	−0.4845	0.1077	0.8878	0.072*	
C46	−0.2898 (7)	0.0702 (3)	0.8368 (3)	0.0460 (14)	
H46A	−0.2500	0.0372	0.8766	0.055*	
C47	0.2758 (9)	0.4882 (4)	0.4682 (4)	0.076 (2)	
H47A	0.2122	0.5311	0.4446	0.091*	
C48	0.2239 (11)	0.4431 (6)	0.5367 (5)	0.099 (3)	
H48A	0.1276	0.4558	0.5591	0.119*	
C49	0.3167 (14)	0.3790 (6)	0.5716 (4)	0.098 (3)	
H49A	0.2835	0.3471	0.6176	0.118*	
C50	0.4554 (11)	0.3634 (4)	0.5381 (4)	0.082 (2)	
H50A	0.5208	0.3208	0.5606	0.098*	
C51	0.4999 (9)	0.4109 (4)	0.4702 (3)	0.0661 (19)	
H51A	0.5968	0.3992	0.4477	0.079*	
C52	−0.0476 (7)	0.1167 (4)	0.4325 (3)	0.0565 (16)	
H52A	0.0274	0.1565	0.4096	0.068*	
C53	−0.0963 (9)	0.0979 (4)	0.5028 (4)	0.0683 (19)	
H53A	−0.0547	0.1244	0.5265	0.082*	
C54	−0.2085 (8)	0.0388 (5)	0.5370 (3)	0.068 (2)	
H54A	−0.2405	0.0231	0.5845	0.081*	
C55	−0.2708 (7)	0.0041 (4)	0.4994 (3)	0.0587 (17)	
H55A	−0.3497	−0.0339	0.5205	0.070*	
C56	−0.2150 (7)	0.0263 (4)	0.4302 (3)	0.0510 (15)	
H56A	−0.2581	0.0019	0.4053	0.061*	

### Atomic displacement parameters (Å^2^)

**Table:**

	*U*^11^	*U*^22^	*U*^33^	*U*^12^	*U*^13^	*U*^23^
Ni1	0.0270 (3)	0.0217 (3)	0.0212 (3)	−0.0014 (3)	−0.0008 (2)	−0.0049 (2)
Ni2	0.0281 (3)	0.0216 (3)	0.0199 (3)	−0.0018 (3)	−0.0014 (2)	−0.0046 (2)
Cl1	0.0371 (7)	0.0439 (8)	0.0326 (7)	0.0084 (6)	−0.0079 (6)	0.0031 (6)
Cl2	0.0268 (7)	0.0482 (8)	0.0355 (7)	0.0012 (6)	0.0020 (5)	−0.0035 (6)
Cl3	0.0268 (7)	0.0483 (8)	0.0369 (7)	0.0000 (6)	0.0011 (5)	−0.0033 (6)
Cl4	0.0359 (7)	0.0489 (8)	0.0351 (7)	0.0088 (6)	−0.0088 (6)	0.0036 (6)
Cl5	0.0479 (8)	0.0466 (8)	0.0386 (8)	0.0115 (7)	0.0124 (6)	−0.0030 (6)
Cl6	0.0462 (8)	0.0490 (8)	0.0353 (7)	0.0161 (7)	−0.0027 (6)	0.0022 (6)
Cl7	0.0439 (8)	0.0490 (8)	0.0342 (7)	0.0140 (7)	0.0074 (6)	−0.0008 (6)
Cl8	0.0415 (8)	0.0504 (8)	0.0335 (7)	0.0112 (7)	−0.0054 (6)	0.0005 (6)
O1	0.0387 (19)	0.0242 (17)	0.0248 (17)	−0.0089 (16)	−0.0023 (14)	−0.0042 (13)
O2	0.044 (2)	0.0299 (18)	0.0251 (18)	−0.0077 (16)	−0.0026 (15)	−0.0090 (14)
O3	0.050 (2)	0.0339 (19)	0.0222 (17)	−0.0120 (17)	−0.0018 (16)	−0.0088 (15)
O4	0.0379 (19)	0.0239 (18)	0.0266 (17)	−0.0061 (16)	−0.0046 (15)	−0.0053 (14)
O5	0.0374 (19)	0.0285 (18)	0.0234 (17)	−0.0066 (16)	0.0012 (15)	−0.0012 (14)
O6	0.066 (3)	0.054 (2)	0.032 (2)	−0.020 (2)	−0.0074 (19)	−0.0110 (18)
O7	0.118 (4)	0.058 (3)	0.029 (2)	−0.039 (3)	0.002 (2)	−0.0134 (19)
O8	0.037 (2)	0.0289 (19)	0.0239 (17)	−0.0030 (16)	0.0015 (15)	−0.0004 (14)
O9	0.0367 (19)	0.0318 (18)	0.0250 (17)	−0.0058 (16)	−0.0018 (15)	−0.0071 (14)
O10	0.0359 (19)	0.0341 (19)	0.0169 (16)	−0.0038 (16)	−0.0029 (14)	−0.0060 (13)
N1	0.027 (2)	0.029 (2)	0.025 (2)	−0.0014 (18)	−0.0019 (17)	−0.0057 (17)
N2	0.028 (2)	0.029 (2)	0.031 (2)	−0.0013 (18)	−0.0015 (18)	−0.0129 (18)
N3	0.031 (2)	0.027 (2)	0.036 (2)	0.0001 (19)	−0.0029 (19)	−0.0105 (18)
N4	0.029 (2)	0.022 (2)	0.022 (2)	−0.0008 (17)	−0.0029 (16)	−0.0055 (16)
N5	0.029 (2)	0.027 (2)	0.025 (2)	−0.0003 (18)	0.0018 (17)	−0.0083 (17)
N6	0.027 (2)	0.034 (2)	0.033 (2)	−0.0025 (19)	0.0043 (18)	−0.0128 (19)
N7	0.066 (4)	0.053 (3)	0.044 (3)	−0.017 (3)	0.005 (3)	−0.019 (3)
N8	0.049 (3)	0.057 (3)	0.044 (3)	0.001 (3)	−0.009 (2)	−0.019 (2)
C1	0.022 (2)	0.028 (3)	0.022 (2)	0.003 (2)	−0.0038 (19)	−0.002 (2)
C2	0.032 (3)	0.022 (2)	0.022 (2)	−0.005 (2)	−0.003 (2)	−0.0085 (19)
C3	0.028 (3)	0.026 (2)	0.026 (2)	−0.001 (2)	−0.007 (2)	−0.007 (2)
C4	0.021 (2)	0.023 (2)	0.032 (3)	−0.005 (2)	−0.004 (2)	−0.007 (2)
C5	0.031 (3)	0.020 (2)	0.024 (2)	−0.006 (2)	−0.005 (2)	−0.0055 (18)
C6	0.030 (3)	0.022 (2)	0.024 (2)	0.000 (2)	−0.008 (2)	−0.0018 (19)
C7	0.020 (2)	0.029 (3)	0.027 (2)	−0.002 (2)	−0.002 (2)	−0.011 (2)
C8	0.024 (2)	0.023 (2)	0.024 (2)	0.001 (2)	−0.001 (2)	−0.0022 (19)
C9	0.035 (3)	0.036 (3)	0.021 (2)	−0.002 (2)	0.000 (2)	−0.006 (2)
C10	0.031 (3)	0.022 (2)	0.027 (3)	−0.006 (2)	0.000 (2)	−0.007 (2)
C11	0.032 (3)	0.017 (2)	0.029 (3)	0.003 (2)	−0.004 (2)	−0.0004 (19)
C12	0.033 (3)	0.022 (2)	0.029 (3)	−0.003 (2)	0.004 (2)	−0.004 (2)
C13	0.035 (3)	0.024 (2)	0.026 (3)	−0.003 (2)	0.002 (2)	−0.009 (2)
C14	0.034 (3)	0.029 (3)	0.030 (3)	−0.004 (2)	−0.006 (2)	−0.003 (2)
C15	0.031 (3)	0.029 (3)	0.020 (2)	0.000 (2)	0.006 (2)	−0.003 (2)
C16	0.042 (3)	0.029 (3)	0.027 (3)	0.000 (2)	−0.001 (2)	−0.005 (2)
C17	0.038 (3)	0.032 (3)	0.035 (3)	−0.001 (2)	−0.005 (2)	−0.015 (2)
C18	0.046 (3)	0.037 (3)	0.046 (3)	0.010 (3)	−0.013 (3)	−0.019 (3)
C19	0.040 (3)	0.054 (4)	0.035 (3)	0.018 (3)	−0.007 (2)	−0.017 (3)
C20	0.032 (3)	0.058 (4)	0.030 (3)	0.000 (3)	−0.002 (2)	−0.004 (3)
C21	0.037 (3)	0.030 (3)	0.027 (3)	−0.004 (2)	−0.005 (2)	−0.002 (2)
C22	0.038 (3)	0.034 (3)	0.037 (3)	−0.003 (2)	−0.012 (2)	−0.012 (2)
C23	0.060 (4)	0.059 (4)	0.039 (3)	−0.006 (3)	−0.009 (3)	−0.020 (3)
C24	0.072 (4)	0.077 (5)	0.044 (4)	−0.021 (4)	−0.006 (3)	−0.041 (3)
C25	0.068 (4)	0.055 (4)	0.053 (4)	−0.021 (3)	0.006 (3)	−0.035 (3)
C26	0.043 (3)	0.030 (3)	0.040 (3)	−0.007 (3)	0.002 (2)	−0.015 (2)
C27	0.045 (3)	0.047 (3)	0.040 (3)	−0.007 (3)	0.005 (3)	−0.017 (3)
C28	0.044 (4)	0.064 (4)	0.069 (4)	−0.002 (3)	0.004 (3)	−0.040 (4)
C29	0.040 (3)	0.039 (3)	0.092 (5)	0.005 (3)	−0.013 (4)	−0.037 (3)
C30	0.047 (4)	0.041 (3)	0.066 (4)	0.008 (3)	−0.026 (3)	−0.017 (3)
C31	0.038 (3)	0.032 (3)	0.044 (3)	0.001 (2)	−0.009 (3)	−0.010 (2)
C32	0.029 (3)	0.026 (3)	0.038 (3)	0.002 (2)	−0.007 (2)	−0.010 (2)
C33	0.041 (3)	0.034 (3)	0.058 (4)	0.009 (3)	−0.013 (3)	−0.023 (3)
C34	0.031 (3)	0.048 (3)	0.052 (3)	0.011 (3)	−0.015 (3)	−0.023 (3)
C35	0.031 (3)	0.038 (3)	0.038 (3)	0.002 (2)	−0.013 (2)	−0.006 (2)
C36	0.034 (3)	0.028 (3)	0.028 (3)	−0.005 (2)	−0.007 (2)	−0.008 (2)
C37	0.032 (3)	0.037 (3)	0.031 (3)	0.001 (2)	−0.004 (2)	−0.016 (2)
C38	0.045 (3)	0.065 (4)	0.034 (3)	0.002 (3)	−0.012 (3)	−0.020 (3)
C39	0.064 (4)	0.074 (5)	0.062 (4)	−0.006 (4)	−0.010 (3)	−0.047 (4)
C40	0.082 (5)	0.042 (3)	0.060 (4)	−0.008 (3)	−0.011 (4)	−0.033 (3)
C41	0.050 (3)	0.031 (3)	0.038 (3)	−0.005 (3)	0.000 (3)	−0.015 (2)
C42	0.040 (3)	0.029 (3)	0.043 (3)	0.000 (2)	−0.013 (2)	−0.008 (2)
C43	0.038 (3)	0.047 (4)	0.068 (4)	0.000 (3)	−0.017 (3)	−0.016 (3)
C44	0.049 (4)	0.041 (4)	0.097 (6)	0.011 (3)	−0.011 (4)	−0.031 (4)
C45	0.046 (4)	0.067 (4)	0.069 (5)	0.000 (3)	0.012 (3)	−0.038 (4)
C46	0.049 (4)	0.050 (4)	0.044 (3)	0.002 (3)	−0.004 (3)	−0.025 (3)
C47	0.075 (5)	0.063 (5)	0.093 (6)	−0.016 (4)	0.001 (5)	−0.034 (4)
C48	0.093 (7)	0.098 (7)	0.114 (8)	−0.049 (6)	0.062 (6)	−0.072 (6)
C49	0.158 (10)	0.083 (6)	0.059 (5)	−0.076 (7)	0.027 (6)	−0.036 (5)
C50	0.128 (7)	0.054 (4)	0.061 (5)	−0.028 (5)	−0.020 (5)	−0.009 (4)
C51	0.072 (5)	0.075 (5)	0.054 (4)	−0.022 (4)	0.003 (4)	−0.027 (4)
C52	0.054 (4)	0.051 (4)	0.070 (4)	0.006 (3)	−0.014 (3)	−0.028 (3)
C53	0.074 (5)	0.078 (5)	0.078 (5)	0.016 (4)	−0.026 (4)	−0.055 (4)
C54	0.069 (5)	0.089 (5)	0.046 (4)	0.032 (4)	−0.012 (4)	−0.031 (4)
C55	0.047 (4)	0.071 (5)	0.053 (4)	0.008 (3)	−0.004 (3)	−0.020 (3)
C56	0.050 (4)	0.061 (4)	0.052 (4)	0.005 (3)	−0.012 (3)	−0.032 (3)

### Geometric parameters (Å, °)

**Table:**

Ni1—O5	2.065 (3)	C19—C20	1.378 (8)
Ni1—O9	2.071 (3)	C19—H19A	0.9300
Ni1—O1	2.086 (3)	C20—C21	1.380 (7)
Ni1—N1	2.094 (4)	C20—H20A	0.9300
Ni1—N3	2.103 (4)	C21—H21A	0.9300
Ni1—N2	2.106 (4)	C22—C23	1.381 (7)
Ni2—O8	2.067 (3)	C22—H22A	0.9300
Ni2—O4^i^	2.094 (3)	C23—C24	1.364 (8)
Ni2—O10	2.095 (3)	C23—H23A	0.9300
Ni2—N4	2.098 (4)	C24—C25	1.372 (8)
Ni2—N6	2.102 (4)	C24—H24A	0.9300
Ni2—N5	2.124 (4)	C25—C26	1.360 (7)
Cl1—C3	1.729 (5)	C25—H25A	0.9300
Cl2—C4	1.729 (5)	C26—H26A	0.9300
Cl3—C7	1.729 (4)	C27—C28	1.380 (8)
Cl4—C6	1.732 (5)	C27—H27A	0.9300
Cl5—C12	1.734 (5)	C28—C29	1.370 (8)
Cl6—C11	1.735 (5)	C28—H28A	0.9300
Cl7—C15	1.729 (5)	C29—C30	1.362 (8)
Cl8—C14	1.729 (5)	C29—H29A	0.9300
O1—C1	1.255 (5)	C30—C31	1.372 (8)
O2—C1	1.241 (5)	C30—H30A	0.9300
O3—C8	1.232 (5)	C31—H31A	0.9300
O4—C8	1.268 (5)	C32—C33	1.380 (7)
O4—Ni2^ii^	2.094 (3)	C32—H32A	0.9300
O5—C9	1.255 (5)	C33—C34	1.370 (7)
O6—C9	1.228 (6)	C33—H33A	0.9300
O7—C16	1.217 (6)	C34—C35	1.379 (7)
O8—C16	1.267 (6)	C34—H34A	0.9300
O9—H9A	0.8500	C35—C36	1.368 (7)
O9—H9B	0.8499	C35—H35A	0.9300
O10—H10A	0.8499	C36—H36A	0.9300
O10—H10B	0.8502	C37—C38	1.384 (7)
N1—C21	1.336 (6)	C37—H37A	0.9300
N1—C17	1.342 (6)	C38—C39	1.356 (8)
N2—C22	1.338 (6)	C38—H38A	0.9300
N2—C26	1.340 (6)	C39—C40	1.381 (8)
N3—C31	1.335 (6)	C39—H39A	0.9300
N3—C27	1.334 (6)	C40—C41	1.366 (7)
N4—C32	1.343 (6)	C40—H40A	0.9300
N4—C36	1.343 (6)	C41—H41A	0.9300
N5—C41	1.324 (6)	C42—C43	1.368 (8)
N5—C37	1.332 (6)	C42—H42A	0.9300
N6—C46	1.332 (6)	C43—C44	1.372 (9)
N6—C42	1.345 (6)	C43—H43A	0.9300
N7—C51	1.325 (8)	C44—C45	1.368 (8)
N7—C47	1.331 (8)	C44—H44A	0.9300
N8—C56	1.330 (7)	C45—C46	1.384 (8)
N8—C52	1.335 (7)	C45—H45A	0.9300
C1—C2	1.533 (6)	C46—H46A	0.9300
C2—C7	1.385 (6)	C47—C48	1.379 (10)
C2—C3	1.389 (6)	C47—H47A	0.9300
C3—C4	1.391 (6)	C48—C49	1.376 (12)
C4—C5	1.387 (6)	C48—H48A	0.9300
C5—C6	1.377 (6)	C49—C50	1.337 (12)
C5—C8	1.523 (6)	C49—H49A	0.9300
C6—C7	1.390 (6)	C50—C51	1.367 (9)
C9—C10	1.542 (6)	C50—H50A	0.9300
C10—C11	1.381 (6)	C51—H51A	0.9300
C10—C15	1.389 (6)	C52—C53	1.384 (9)
C11—C12	1.395 (6)	C52—H52A	0.9300
C12—C13	1.379 (6)	C53—C54	1.386 (9)
C13—C14	1.384 (7)	C53—H53A	0.9300
C13—C16	1.539 (6)	C54—C55	1.363 (9)
C14—C15	1.386 (6)	C54—H54A	0.9300
C17—C18	1.369 (7)	C55—C56	1.369 (8)
C17—H17A	0.9300	C55—H55A	0.9300
C18—C19	1.372 (7)	C56—H56A	0.9300
C18—H18A	0.9300		
			
O5—Ni1—O9	85.31 (12)	C18—C19—C20	118.7 (5)
O5—Ni1—O1	174.04 (13)	C18—C19—H19A	120.6
O9—Ni1—O1	88.76 (12)	C20—C19—H19A	120.6
O5—Ni1—N1	88.98 (13)	C19—C20—C21	118.7 (5)
O9—Ni1—N1	91.83 (14)	C19—C20—H20A	120.7
O1—Ni1—N1	91.79 (13)	C21—C20—H20A	120.7
O5—Ni1—N3	91.68 (14)	N1—C21—C20	123.1 (5)
O9—Ni1—N3	86.30 (14)	N1—C21—H21A	118.4
O1—Ni1—N3	87.36 (14)	C20—C21—H21A	118.4
N1—Ni1—N3	177.96 (15)	N2—C22—C23	123.6 (5)
O5—Ni1—N2	101.70 (13)	N2—C22—H22A	118.2
O9—Ni1—N2	172.48 (13)	C23—C22—H22A	118.2
O1—Ni1—N2	84.19 (13)	C24—C23—C22	118.6 (6)
N1—Ni1—N2	90.99 (15)	C24—C23—H23A	120.7
N3—Ni1—N2	90.76 (15)	C22—C23—H23A	120.7
O8—Ni2—O4^i^	173.56 (13)	C23—C24—C25	118.3 (5)
O8—Ni2—O10	84.59 (12)	C23—C24—H24A	120.8
O4^i^—Ni2—O10	89.13 (12)	C25—C24—H24A	120.8
O8—Ni2—N4	89.24 (13)	C26—C25—C24	120.0 (6)
O4^i^—Ni2—N4	92.32 (13)	C26—C25—H25A	120.0
O10—Ni2—N4	91.11 (13)	C24—C25—H25A	120.0
O8—Ni2—N6	90.75 (14)	N2—C26—C25	122.9 (5)
O4^i^—Ni2—N6	87.57 (14)	N2—C26—H26A	118.5
O10—Ni2—N6	87.79 (14)	C25—C26—H26A	118.5
N4—Ni2—N6	178.89 (15)	N3—C27—C28	122.6 (6)
O8—Ni2—N5	103.27 (13)	N3—C27—H27A	118.7
O4^i^—Ni2—N5	82.98 (13)	C28—C27—H27A	118.7
O10—Ni2—N5	172.07 (12)	C29—C28—C27	119.0 (6)
N4—Ni2—N5	90.03 (15)	C29—C28—H28A	120.5
N6—Ni2—N5	91.05 (15)	C27—C28—H28A	120.5
C1—O1—Ni1	132.2 (3)	C30—C29—C28	119.0 (6)
C8—O4—Ni2^ii^	130.6 (3)	C30—C29—H29A	120.5
C9—O5—Ni1	141.5 (3)	C28—C29—H29A	120.5
C16—O8—Ni2	136.5 (3)	C29—C30—C31	118.9 (6)
Ni1—O9—H9A	114.1	C29—C30—H30A	120.6
Ni1—O9—H9B	126.7	C31—C30—H30A	120.6
H9A—O9—H9B	95.8	N3—C31—C30	123.3 (5)
Ni2—O10—H10A	128.5	N3—C31—H31A	118.4
Ni2—O10—H10B	95.6	C30—C31—H31A	118.4
H10A—O10—H10B	114.5	N4—C32—C33	122.8 (4)
C21—N1—C17	117.2 (4)	N4—C32—H32A	118.6
C21—N1—Ni1	120.1 (3)	C33—C32—H32A	118.6
C17—N1—Ni1	122.7 (3)	C34—C33—C32	120.1 (5)
C22—N2—C26	116.4 (4)	C34—C33—H33A	119.9
C22—N2—Ni1	120.6 (3)	C32—C33—H33A	119.9
C26—N2—Ni1	122.5 (3)	C33—C34—C35	117.5 (5)
C31—N3—C27	117.3 (5)	C33—C34—H34A	121.3
C31—N3—Ni1	120.4 (4)	C35—C34—H34A	121.3
C27—N3—Ni1	122.3 (4)	C36—C35—C34	119.6 (5)
C32—N4—C36	116.4 (4)	C36—C35—H35A	120.2
C32—N4—Ni2	123.4 (3)	C34—C35—H35A	120.2
C36—N4—Ni2	120.1 (3)	N4—C36—C35	123.6 (5)
C41—N5—C37	116.8 (4)	N4—C36—H36A	118.2
C41—N5—Ni2	123.5 (3)	C35—C36—H36A	118.2
C37—N5—Ni2	119.3 (3)	N5—C37—C38	123.3 (5)
C46—N6—C42	116.8 (5)	N5—C37—H37A	118.4
C46—N6—Ni2	122.2 (4)	C38—C37—H37A	118.4
C42—N6—Ni2	121.0 (3)	C39—C38—C37	118.7 (5)
C51—N7—C47	116.7 (6)	C39—C38—H38A	120.6
C56—N8—C52	116.5 (5)	C37—C38—H38A	120.6
O2—C1—O1	129.3 (4)	C38—C39—C40	118.7 (5)
O2—C1—C2	117.0 (4)	C38—C39—H39A	120.7
O1—C1—C2	113.6 (4)	C40—C39—H39A	120.7
C7—C2—C3	118.6 (4)	C41—C40—C39	118.7 (6)
C7—C2—C1	120.9 (4)	C41—C40—H40A	120.6
C3—C2—C1	120.5 (4)	C39—C40—H40A	120.6
C2—C3—C4	121.0 (4)	N5—C41—C40	123.7 (5)
C2—C3—Cl1	118.7 (3)	N5—C41—H41A	118.1
C4—C3—Cl1	120.3 (4)	C40—C41—H41A	118.1
C5—C4—C3	120.1 (4)	N6—C42—C43	122.9 (5)
C5—C4—Cl2	119.7 (3)	N6—C42—H42A	118.5
C3—C4—Cl2	120.2 (4)	C43—C42—H42A	118.5
C6—C5—C4	118.8 (4)	C42—C43—C44	119.4 (6)
C6—C5—C8	120.7 (4)	C42—C43—H43A	120.3
C4—C5—C8	120.5 (4)	C44—C43—H43A	120.3
C5—C6—C7	121.3 (4)	C45—C44—C43	118.9 (6)
C5—C6—Cl4	118.5 (3)	C45—C44—H44A	120.6
C7—C6—Cl4	120.2 (4)	C43—C44—H44A	120.6
C2—C7—C6	120.2 (4)	C44—C45—C46	118.5 (6)
C2—C7—Cl3	119.4 (3)	C44—C45—H45A	120.8
C6—C7—Cl3	120.5 (4)	C46—C45—H45A	120.8
O3—C8—O4	128.4 (4)	N6—C46—C45	123.5 (6)
O3—C8—C5	117.1 (4)	N6—C46—H46A	118.2
O4—C8—C5	114.5 (4)	C45—C46—H46A	118.2
O6—C9—O5	130.0 (4)	N7—C47—C48	122.4 (8)
O6—C9—C10	116.1 (4)	N7—C47—H47A	118.8
O5—C9—C10	113.9 (4)	C48—C47—H47A	118.8
C11—C10—C15	117.3 (4)	C49—C48—C47	119.0 (8)
C11—C10—C9	122.0 (4)	C49—C48—H48A	120.5
C15—C10—C9	120.6 (4)	C47—C48—H48A	120.5
C10—C11—C12	121.5 (4)	C50—C49—C48	118.8 (8)
C10—C11—Cl6	119.0 (3)	C50—C49—H49A	120.6
C12—C11—Cl6	119.5 (4)	C48—C49—H49A	120.6
C13—C12—C11	121.0 (4)	C49—C50—C51	119.2 (9)
C13—C12—Cl5	118.8 (3)	C49—C50—H50A	120.4
C11—C12—Cl5	120.2 (4)	C51—C50—H50A	120.4
C12—C13—C14	117.5 (4)	N7—C51—C50	124.0 (7)
C12—C13—C16	122.2 (4)	N7—C51—H51A	118.0
C14—C13—C16	120.3 (4)	C50—C51—H51A	118.0
C13—C14—C15	121.6 (4)	N8—C52—C53	123.1 (6)
C13—C14—Cl8	118.9 (4)	N8—C52—H52A	118.5
C15—C14—Cl8	119.5 (4)	C53—C52—H52A	118.5
C14—C15—C10	121.0 (4)	C52—C53—C54	118.6 (6)
C14—C15—Cl7	120.3 (4)	C52—C53—H53A	120.7
C10—C15—Cl7	118.7 (3)	C54—C53—H53A	120.7
O7—C16—O8	128.7 (5)	C55—C54—C53	118.5 (6)
O7—C16—C13	117.9 (4)	C55—C54—H54A	120.7
O8—C16—C13	113.4 (4)	C53—C54—H54A	120.7
N1—C17—C18	123.0 (5)	C54—C55—C56	118.7 (6)
N1—C17—H17A	118.5	C54—C55—H55A	120.6
C18—C17—H17A	118.5	C56—C55—H55A	120.6
C17—C18—C19	119.3 (5)	N8—C56—C55	124.5 (6)
C17—C18—H18A	120.4	N8—C56—H56A	117.8
C19—C18—H18A	120.4	C55—C56—H56A	117.8
			
O9—Ni1—O1—C1	−13.3 (4)	Ni1—O5—C9—O6	7.9 (10)
N1—Ni1—O1—C1	78.5 (4)	Ni1—O5—C9—C10	−171.8 (3)
N3—Ni1—O1—C1	−99.7 (4)	O6—C9—C10—C11	−84.6 (6)
N2—Ni1—O1—C1	169.3 (4)	O5—C9—C10—C11	95.2 (6)
O9—Ni1—O5—C9	179.4 (6)	O6—C9—C10—C15	94.4 (6)
N1—Ni1—O5—C9	87.5 (5)	O5—C9—C10—C15	−85.8 (6)
N3—Ni1—O5—C9	−94.4 (6)	C15—C10—C11—C12	−0.9 (7)
N2—Ni1—O5—C9	−3.3 (6)	C9—C10—C11—C12	178.1 (4)
O10—Ni2—O8—C16	170.9 (5)	C15—C10—C11—Cl6	178.7 (3)
N4—Ni2—O8—C16	−98.0 (5)	C9—C10—C11—Cl6	−2.3 (6)
N6—Ni2—O8—C16	83.2 (5)	C10—C11—C12—C13	1.1 (7)
N5—Ni2—O8—C16	−8.1 (5)	Cl6—C11—C12—C13	−178.5 (4)
O5—Ni1—N1—C21	−52.0 (3)	C10—C11—C12—Cl5	179.6 (4)
O9—Ni1—N1—C21	−137.3 (3)	Cl6—C11—C12—Cl5	0.0 (6)
O1—Ni1—N1—C21	133.9 (3)	C11—C12—C13—C14	0.0 (7)
N2—Ni1—N1—C21	49.6 (3)	Cl5—C12—C13—C14	−178.5 (4)
O5—Ni1—N1—C17	126.8 (4)	C11—C12—C13—C16	177.5 (4)
O9—Ni1—N1—C17	41.6 (3)	Cl5—C12—C13—C16	−1.0 (6)
O1—Ni1—N1—C17	−47.3 (4)	C12—C13—C14—C15	−1.2 (7)
N2—Ni1—N1—C17	−131.5 (4)	C16—C13—C14—C15	−178.8 (4)
O5—Ni1—N2—C22	143.2 (4)	C12—C13—C14—Cl8	176.6 (4)
O1—Ni1—N2—C22	−37.6 (4)	C16—C13—C14—Cl8	−1.0 (6)
N1—Ni1—N2—C22	54.1 (4)	C13—C14—C15—C10	1.4 (7)
N3—Ni1—N2—C22	−124.9 (4)	Cl8—C14—C15—C10	−176.4 (4)
O5—Ni1—N2—C26	−44.9 (4)	C13—C14—C15—Cl7	−178.5 (4)
O1—Ni1—N2—C26	134.2 (4)	Cl8—C14—C15—Cl7	3.7 (6)
N1—Ni1—N2—C26	−134.1 (4)	C11—C10—C15—C14	−0.3 (7)
N3—Ni1—N2—C26	47.0 (4)	C9—C10—C15—C14	−179.3 (4)
O5—Ni1—N3—C31	−33.3 (4)	C11—C10—C15—Cl7	179.6 (4)
O9—Ni1—N3—C31	51.9 (4)	C9—C10—C15—Cl7	0.6 (6)
O1—Ni1—N3—C31	140.8 (4)	Ni2—O8—C16—O7	1.6 (10)
N2—Ni1—N3—C31	−135.0 (4)	Ni2—O8—C16—C13	179.7 (3)
O5—Ni1—N3—C27	149.4 (4)	C12—C13—C16—O7	−87.4 (7)
O9—Ni1—N3—C27	−125.5 (4)	C14—C13—C16—O7	90.1 (6)
O1—Ni1—N3—C27	−36.5 (4)	C12—C13—C16—O8	94.3 (6)
N2—Ni1—N3—C27	47.6 (4)	C14—C13—C16—O8	−88.2 (6)
O8—Ni2—N4—C32	−124.6 (4)	C21—N1—C17—C18	0.3 (7)
O4^i^—Ni2—N4—C32	49.2 (4)	Ni1—N1—C17—C18	−178.6 (4)
O10—Ni2—N4—C32	−40.0 (3)	N1—C17—C18—C19	−1.5 (8)
N5—Ni2—N4—C32	132.2 (3)	C17—C18—C19—C20	2.0 (8)
O8—Ni2—N4—C36	51.2 (3)	C18—C19—C20—C21	−1.3 (7)
O4^i^—Ni2—N4—C36	−135.1 (3)	C17—N1—C21—C20	0.4 (7)
O10—Ni2—N4—C36	135.7 (3)	Ni1—N1—C21—C20	179.3 (4)
N5—Ni2—N4—C36	−52.1 (3)	C19—C20—C21—N1	0.1 (8)
O8—Ni2—N5—C41	48.2 (4)	C26—N2—C22—C23	−2.9 (7)
O4^i^—Ni2—N5—C41	−130.3 (4)	Ni1—N2—C22—C23	169.4 (4)
N4—Ni2—N5—C41	137.4 (4)	N2—C22—C23—C24	0.6 (9)
N6—Ni2—N5—C41	−42.8 (4)	C22—C23—C24—C25	1.2 (9)
O8—Ni2—N5—C37	−139.9 (3)	C23—C24—C25—C26	−0.6 (9)
O4^i^—Ni2—N5—C37	41.7 (3)	C22—N2—C26—C25	3.6 (7)
N4—Ni2—N5—C37	−50.6 (3)	Ni1—N2—C26—C25	−168.6 (4)
N6—Ni2—N5—C37	129.1 (3)	C24—C25—C26—N2	−2.0 (9)
O8—Ni2—N6—C46	−150.5 (4)	C31—N3—C27—C28	−0.7 (8)
O4^i^—Ni2—N6—C46	35.8 (4)	Ni1—N3—C27—C28	176.8 (4)
O10—Ni2—N6—C46	125.0 (4)	N3—C27—C28—C29	0.9 (9)
N5—Ni2—N6—C46	−47.2 (4)	C27—C28—C29—C30	−1.1 (8)
O8—Ni2—N6—C42	30.5 (4)	C28—C29—C30—C31	1.0 (8)
O4^i^—Ni2—N6—C42	−143.3 (4)	C27—N3—C31—C30	0.6 (7)
O10—Ni2—N6—C42	−54.1 (3)	Ni1—N3—C31—C30	−176.9 (4)
N5—Ni2—N6—C42	133.8 (4)	C29—C30—C31—N3	−0.8 (8)
Ni1—O1—C1—O2	−1.3 (8)	C36—N4—C32—C33	−0.4 (7)
Ni1—O1—C1—C2	180.0 (3)	Ni2—N4—C32—C33	175.4 (4)
O2—C1—C2—C7	−99.7 (5)	N4—C32—C33—C34	0.6 (8)
O1—C1—C2—C7	79.2 (6)	C32—C33—C34—C35	0.3 (8)
O2—C1—C2—C3	82.9 (6)	C33—C34—C35—C36	−1.3 (8)
O1—C1—C2—C3	−98.1 (5)	C32—N4—C36—C35	−0.7 (7)
C7—C2—C3—C4	2.3 (7)	Ni2—N4—C36—C35	−176.7 (4)
C1—C2—C3—C4	179.7 (4)	C34—C35—C36—N4	1.6 (8)
C7—C2—C3—Cl1	−178.0 (3)	C41—N5—C37—C38	1.5 (7)
C1—C2—C3—Cl1	−0.6 (6)	Ni2—N5—C37—C38	−171.0 (4)
C2—C3—C4—C5	−2.3 (7)	N5—C37—C38—C39	0.5 (8)
Cl1—C3—C4—C5	178.0 (4)	C37—C38—C39—C40	−1.3 (9)
C2—C3—C4—Cl2	178.0 (4)	C38—C39—C40—C41	0.3 (9)
Cl1—C3—C4—Cl2	−1.7 (6)	C37—N5—C41—C40	−2.7 (7)
C3—C4—C5—C6	1.9 (7)	Ni2—N5—C41—C40	169.5 (4)
Cl2—C4—C5—C6	−178.3 (4)	C39—C40—C41—N5	1.9 (9)
C3—C4—C5—C8	−177.1 (4)	C46—N6—C42—C43	1.5 (7)
Cl2—C4—C5—C8	2.6 (6)	Ni2—N6—C42—C43	−179.4 (4)
C4—C5—C6—C7	−1.7 (7)	N6—C42—C43—C44	−0.7 (8)
C8—C5—C6—C7	177.4 (4)	C42—C43—C44—C45	−0.5 (9)
C4—C5—C6—Cl4	179.3 (3)	C43—C44—C45—C46	0.8 (9)
C8—C5—C6—Cl4	−1.6 (6)	C42—N6—C46—C45	−1.1 (8)
C3—C2—C7—C6	−2.0 (7)	Ni2—N6—C46—C45	179.8 (4)
C1—C2—C7—C6	−179.4 (4)	C44—C45—C46—N6	0.0 (9)
C3—C2—C7—Cl3	177.9 (3)	C51—N7—C47—C48	0.1 (10)
C1—C2—C7—Cl3	0.5 (6)	N7—C47—C48—C49	−0.8 (12)
C5—C6—C7—C2	1.7 (7)	C47—C48—C49—C50	1.0 (12)
Cl4—C6—C7—C2	−179.3 (4)	C48—C49—C50—C51	−0.7 (12)
C5—C6—C7—Cl3	−178.1 (4)	C47—N7—C51—C50	0.3 (10)
Cl4—C6—C7—Cl3	0.8 (6)	C49—C50—C51—N7	0.0 (11)
Ni2^ii^—O4—C8—O3	3.1 (8)	C56—N8—C52—C53	2.7 (9)
Ni2^ii^—O4—C8—C5	−178.7 (3)	N8—C52—C53—C54	−0.2 (10)
C6—C5—C8—O3	−80.2 (6)	C52—C53—C54—C55	−2.7 (10)
C4—C5—C8—O3	98.8 (5)	C53—C54—C55—C56	3.0 (10)
C6—C5—C8—O4	101.4 (5)	C52—N8—C56—C55	−2.4 (9)
C4—C5—C8—O4	−79.5 (6)	C54—C55—C56—N8	−0.4 (10)

Symmetry codes: (i) *x*−1, *y*−1, *z*+1; (ii) *x*+1, *y*+1, *z*−1.

### Hydrogen-bond geometry (Å, °)

**Table:**

*D*—H···*A*	*D*—H	H···*A*	*D*···*A*	*D*—H···*A*
O9—H9A···O2	0.85	2.02	2.751 (4)	143
O9—H9B···N7	0.85	1.89	2.699 (6)	159
O10—H10A···N8^iii^	0.85	1.97	2.783 (6)	161
O10—H10B···O3^i^	0.85	1.83	2.677 (4)	174

Symmetry codes: (iii) −*x*, −*y*, −*z*+1; (i) *x*−1, *y*−1, *z*+1.

## References

[bb1] Bruker (2005). *APEX2* and *SAINT* Bruker AXS Inc., Madison, Wisconsin, USA.

[bb2] Go, Y. B., Wang, X. Q., Anokhina, E. V. & Jacobson, A. J. (2004). *Inorg. Chem.* **43**, 5360–5367. 10.1021/ic049341u 15310214

[bb3] Kim, J. C., Jo, H., Lough, A. J., Cho, J., Lee, U. & Pyun, S. Y. (2003). *Inorg. Chem. Commun.* **6**, 474–477.

[bb4] Li, Y. G., Hao, N., Lu, Y., Wang, E. B., Kang, Z. H. & Hu, C. J. (2003). *Inorg. Chem.* **42**, 3119–3124. 10.1021/ic026306j 12716210

[bb5] Mulder, F. M., Dingemans, T. J., Wagemake, M. & Kearley, G. J. (2005). *Chem. Phys.* **317**, 113–118.

[bb11] Sheldrick, G. M. (2008*a*). *SADABS.* University of Göttingen, Germany.

[bb6] Sheldrick, G. M. (2008*b*). *Acta Cryst.* A**64**, 112–122. 10.1107/S0108767307043930 18156677

[bb7] Spek, A. L. (2009). *Acta Cryst.* D**65**, 148–155. 10.1107/S090744490804362X PMC2631630 19171970

[bb8] Wang, L. Y., Liu, Z. L., Liao, D. Z., Jiang, Z. H. & Yan, S. P. (2003). *Inorg. Chem. Commun.* **6**, 630–633.

[bb9] Zheng, C. G., Li, S., Zhang, P. P. & Wang, W. X. (2009). *Transition Met. Chem.* **34**, 815–820.

[bb10] Zheng, C.-G., Zhang, J., Hong, J.-Q. & Li, S. (2008). *Acta Cryst.* E**64**, m965. 10.1107/S1600536808018977 PMC2961727 21202811

